# Barrett’s esophagus and the risk of obstructive sleep apnea: a case–control study

**DOI:** 10.1186/1471-230X-13-82

**Published:** 2013-05-11

**Authors:** Linda C Cummings, Ninad Shah, Santo Maimone, Wajeeh Salah, Vijay Khiani, Amitabh Chak

**Affiliations:** 1 Division of Gastroenterology and Liver Disease, Department of Medicine, University Hospitals Case Medical Center, 1100 Euclid Avenue Mailstop 5066, Cleveland, OH, 44106-5066, USA; 2 Gastroenterology Associates, 1400 N Ritter Avenue Suite 370, Indianapolis, IN, 46219, USA; 3 Mayo Clinic Jacksonville, 4500 San Pablo Road, Jacksonville, FL, 32224, USA; 4 University of Illinois at Chicago, Section of Digestive Diseases and Nutrition, 840 South Wood Street (MC716), Chicago, IL, 60612, USA

**Keywords:** Barrett’s esophagus, Gastroesophageal reflux disease, Obesity, Obstructive sleep apnea

## Abstract

**Background:**

Prior studies suggest that obstructive sleep apnea may be associated with gastroesophageal reflux disease, a strong risk factor for Barrett’s esophagus. The goals of this pilot case–control study were to determine whether Barrett’s esophagus patients have an increased likelihood of obstructive sleep apnea and to determine whether nocturnal gastroesophageal reflux symptoms affect the relationship between Barrett’s esophagus and obstructive sleep apnea risk.

**Methods:**

Patients with Barrett’s esophagus completed the Berlin Questionnaire, a validated survey instrument identifying subjects at high risk for obstructive sleep apnea. Two outpatient control groups were recruited: 1) EGD Group, subjects matched to Barrett’s esophagus cases by age, race, and gender with esophagogastroduodenoscopy negative for Barrett’s esophagus; and 2) Colonoscopy Group, patients getting colonoscopy. Rates of scoring at high risk for obstructive sleep apnea were compared. Respondents were also questioned regarding severity of their typical gastroesophageal reflux symptoms and presence of nocturnal gastroesophageal reflux symptoms.

**Results:**

The study included 287 patients (54 Barrett’s esophagus, 62 EGD, and 171 colonoscopy subjects). Barrett’s esophagus patients were slightly older than colonoscopy patients and more obese. 56% (n = 30) of Barrett’s esophagus subjects scored at high risk for obstructive sleep apnea, compared with 42% (n = 26) of EGD subjects (OR 1.73, 95% CI [0.83, 3.62]) and 37% (n = 64) of colonoscopy patients (OR 2.08, 95% CI [1.12, 3.88]). The association between Barrett’s esophagus and scoring at high risk for obstructive sleep apnea compared with colonoscopy patients disappeared after adjusting for age. Barrett’s esophagus patients reported more severe typical heartburn and regurgitation symptoms than either control group. Among all subjects, patients with nocturnal reflux symptoms were more likely to score at high risk for obstructive sleep apnea than patients without nocturnal reflux.

**Conclusions:**

In this pilot study, a high proportion of Barrett’s esophagus subjects scored at high risk for obstructive sleep apnea. Having Barrett’s esophagus was associated with more severe gastroesophageal reflux symptoms, and nocturnal reflux symptoms were associated with scoring at high risk for obstructive sleep apnea. The need for obstructive sleep apnea screening in Barrett’s esophagus patients with nocturnal gastroesophageal reflux symptoms should be further evaluated.

## Background

Esophageal adenocarcinoma (EAC) has dramatically increased in incidence in the United States over the last 3 decades [1,2] and is strongly linked to its precursor metaplastic lesion, Barrett’s esophagus (BE), and gastroesophageal reflux disease (GERD) [3]. Current guidelines recommend considering endoscopic screening of patients with multiple risk factors for Barrett’s esophagus including chronic GERD symptoms [4]. Studies suggest that 8%-13% of patients with GERD have BE [5,6], compared with a rate of 1%-6% in patients without heartburn symptoms [6,7]. The risk of BE increases with increasing duration of reflux symptoms, particularly nocturnal symptoms [3,8]. Moreover, GERD patients with BE typically have a greater duration of esophageal acid exposure than those without BE.

BE and esophageal adenocarcinoma are also associated with obesity [9-11]. The increase in obesity in the United States over the past several decades has been proposed as an explanation for the rising incidence of esophageal adenocarcinoma. Obesity and its associated health conditions represent a growing public health concern in the United States. Obesity-associated obstructive sleep apnea (OSA), for example, is an important albeit underdiagnosed syndrome with detrimental cardiovascular and neurocognitive effects. Like those at risk for BE, patients at risk for OSA are typically male, obese, and middle-aged.

Prior theory and evidence suggest that OSA is associated with gastroesophageal reflux disease. It has been theorized that negative intrapleural pressures generated during nocturnal airway obstruction in OSA could contribute to nocturnal gastroesophageal reflux [12,13], although studies have not demonstrated a direct relationship between obstructive events and nocturnal GERD events [12,14]. Alternatively, it is possible that obstructive events could lead to weakening at the lower esophageal sphincter over time [14]. A population-based study reported increased nocturnal GERD symptoms in patients with sleep-disordered breathing symptoms [15]. A large cohort study suggested that nocturnal GERD may be associated with increased body mass index and sleep complaints [16]. A study of 1093 OSA patients found that frequent nocturnal GERD was associated with more severe OSA, independent of body mass index [13]. Other studies have demonstrated that treatment of OSA with continuous positive airway pressure effectively treats nocturnal gastroesophageal reflux symptoms [17,18]. Continuous positive airway pressure treatment in OSA patients with GERD significantly reduced nocturnal acid contact time as measured by esophageal pH monitoring [18]. Metabolic changes associated with OSA including increased oxidative stress [19] and alteration in serum levels of insulin and insulin-like growth factors [20] could predispose to BE. The role of nocturnal reflux in Barrett’s esophagus needs further investigation. Although some studies have suggested that nocturnal reflux symptoms are not associated with Barrett’s esophagus among GERD patients [21,22], other studies have measured increased nocturnal esophageal acid exposure [23] or nocturnal non-acid reflux [24] in BE patients compared with GERD patients without BE.

Given the growing body of evidence associating OSA with gastroesophageal reflux disease, this pilot study aimed to determine whether patients with BE are more likely to be at risk for OSA. We hypothesized that BE patients would be more likely to score at high risk for OSA on a survey instrument for OSA screening compared with controls. We also aimed to determine if nocturnal gastroesophageal reflux symptoms affected the relationship between BE and high risk for OSA.

## Methods

The study was approved by the University Hospitals Case Medical Center Institutional Review Board and was performed in accordance with the Declaration of Helsinki. We performed a pilot case–control study comparing risk for OSA between subjects with Barrett’s esophagus and 2 control groups. Risk for OSA was determined based on an addended version of the Berlin Questionnaire, a validated survey instrument used for OSA screening that identifies subjects at high risk for OSA [25,26]. Two control groups identified from patients undergoing outpatient endoscopy were recruited including patients from another study as previously described [27]: 1) Subjects matched to BE cases by age ± 5 years, race, and gender with upper endoscopy negative for BE (hereafter referred to as the EGD group); and 2) Patients presenting for outpatient colonoscopy, including open-access cases, who had no prior upper endoscopy at our facility (hereafter referred to as the COL group). If possible, we matched up to 3 EGD control subjects per BE case subject by age (at time of survey) ± 5 years, race, and gender. The survey was distributed via mailings to BE patients who had previously undergone upper endoscopy and in person at the time of endoscopy. The vast majority of control subjects were recruited at the time of endoscopy, with 2 EGD control subjects completing their surveys < 14 days after an upper endoscopy negative for BE. Informed consent was obtained from all subjects.

### Inclusion/exclusion criteria

Barrett’s esophagus cases included patients with a known diagnosis of histologically confirmed short-segment or long-segment Barrett’s esophagus followed for surveillance in our endoscopy lab as well as incident BE cases diagnosed at the time of survey completion based on endoscopic and histological findings. An existing Barrett’s esophagus database was used to identify potential cases. In addition, the results of a pathology database search for intestinal metaplasia were used in conjunction with endoscopy reports to identify potential cases; for these purposes, intestinal metaplasia had to be present on ≥1 biopsy of salmon-colored mucosa from the distal esophagus. We excluded patients with a known diagnosis of obstructive sleep apnea because it was felt that the results of the Berlin Questionnaire might be difficult to interpret in OSA patients compliant with therapy; additionally, the control groups included a subset of patients from another study [27] in which those with OSA were excluded up front and therefore did not complete the Berlin Questionnaire. We excluded patients with esophageal adenocarcinoma, patients aged <18 years, those unable to give consent, and those unable to read or respond to survey questions. In addition, patients with malignancy with distant metastases or those with malignancy who had undergone radiation or chemotherapy within 1 year prior to the survey were excluded. Electronic medical records were reviewed for documentation of metastatic disease and radiation or chemotherapy treatment. Surveys were administered between September 2006 and May 2008.

### Survey Instrument

The Berlin Questionnaire (BQ) is a brief survey that has been validated as a means of identifying patients that are at risk for OSA [25]. This questionnaire asks about weight change, daytime sleepiness, snoring, and presence or absence of hypertension. Based on their responses, subjects are considered to be at high risk for OSA if they meet requirements for at least 2 of 3 symptom categories: 1) persistent symptoms (>3 to 4 times/week) in 2 or more questions about snoring; 2) persistent (>3 to 4 times/week) daytime somnolence and/or drowsiness while driving; and 3) body mass index (BMI) of more than 30 kg/m^2^ (based on self-reported height and weight in this study) or history of high blood pressure. In a primary care setting, being identified by the Berlin Questionnaire to be at high risk for OSA predicted a respiratory disturbance index (RDI) greater than 5 with a sensitivity of 0.86, a specificity of 0.77, a positive predictive value of 0.89, and a likelihood ratio of 3.79 [25]. The Berlin Questionnaire has also been validated in surgical patients [28].

To better assess risk factors, the Berlin Questionnaire was addended with standardized questions regarding the severity of the subject’s typical GERD symptoms (see subsection Summary of Modified Berlin Questionnaire) and presence of nocturnal regurgitation and heartburn. Heartburn or regurgitation symptoms were considered present if the respondent rated his/her usual heartburn or acid regurgitation symptoms as at least mild in severity. Severe heartburn or acid regurgitation symptoms were defined as lifestyle-affecting. Nocturnal gastroesophageal reflux symptoms were considered present if the respondent reported that heartburn or acid regurgitation had awakened him/her in the past year. Nocturnal regurgitation was imputed as absent for 1 COL patient who did not answer the question regarding awakening by nocturnal regurgitation in the past year, but rated his usual acid regurgitation symptoms as nonexistent.

### Summary of Modified Berlin Questionnaire

A summary of the modified Berlin Questionnaire used in the study, including the Berlin Questionnaire and its scoring system and additional questions regarding gastroesophageal reflux disease symptoms, is presented below.

Subjects were considered to be at high risk for obstructive sleep apnea if ≥2 of 3 symptom categories were positive. Categories 1 and 2 were considered positive if the patient answered yes to ≥2 questions, and Category 3 was considered positive if the patient answered yes to ≥1 question.

#### **Category 1: Snoring**

•Does the patient snore?

•Does the patient snore ≥3-4 times per week?

•Does the patient stop breathing while sleeping ≥3-4 times per week?

•Is the patient’s snoring at least louder than talking?

•Does the snoring bother other people?

#### **Category 2: Daytime Somnolence**

•Does the patient feel tired after sleeping ≥3-4 times per week?

•Does the patient feel tired during waketime ≥3-4 times per week?

•Has the patient ever fallen asleep while driving?

•Does falling asleep while driving occur ≥3-4 times per week?

#### **Category 3: Obesity/Hypertension**

•Does the patient have high blood pressure?

•Does the patient have a body mass index >30? (Based on self-reported height and weight)

### **Questions regarding Gastroesophageal Reflux Disease**

#### **Questions about heartburn**

•Severity rating of the patient’s typical heartburn (nonexistent, mild, moderate, or severe)

•Has heartburn awakened the patient at night in the past year?

#### **Questions about acid regurgitation**

•Severity rating of the patient’s typical acid regurgitation (nonexistent, mild, moderate, or severe)

•Has acid regurgitation awakened the patient at night in the past year?

### Power calculation/Study population

We anticipated based on our population of approximately 150 BE patients that are regularly followed at our Digestive Health Institute and an expected survey response rate of 50% that we would be able to capture 75 BE cases. Prior studies in the United States using the Berlin Questionnaire in primary care settings and a cardiology referral clinic found that 32%-36% of subjects were identified as being at high risk for OSA [26,29]. Assuming a 35% prevalence of being at high risk for OSA (hereafter referred to as BQ+) in the control group and a 55% prevalence of being BQ+ in our case group, our study would have 83% power to detect this difference at α = 0.05. Of note, our study findings were consistent with the incidences arrived at in our power calculation. This calculation did not account for matching, which should increase the power of the study.

We recruited 73 Barrett’s esophagus cases and 242 controls, of which 66 were in the EGD group and 176 were in the COL group. The final comparison groups were comprised of 54 Barrett’s esophagus cases, 62 EGD control patients, and 171 COL control patients after the following exclusions: 6 BE cases with esophageal adenocarcinoma; 4 subjects with recent chemotherapy or radiation for malignancy (1 BE case, 2 EGD patients, and 1 COL patient); 4 COL patients with metastatic disease; 11 BE cases previously diagnosed with OSA (n = 10) or missing OSA status (n = 1); 2 EGD patients previously diagnosed with OSA; and 1 patient with missing case status.

### Statistical considerations

Descriptive statistics were performed on baseline characteristics. Continuous variables were compared between control and case groups using t-tests. Categorical variables were compared between control and case groups using chi-square tests or Fisher’s exact test where appropriate. Covariates that significantly differed between the COL and BE groups were evaluated further using a univariate logistic regression model to identify predictors of scoring at high risk for OSA on the Berlin Questionnaire. A multivariate logistic regression model was developed including covariates that were significant predictors in univariate models by the likelihood ratio test. Data were analyzed using SAS (9.2 for Windows, SAS Institute Inc., Cary, North Carolina, USA).

### Sensitivity Analysis

To account for possible misclassification of Barrett’s esophagus patients as controls in the COL group, a sensitivity analysis was performed assuming a 7% rate of misclassification based on prior literature [6]. The odds ratio describing the association between BE and being BQ+ was calculated based on different assumptions regarding the BQ+ rate among these misclassified patients.

## Results

**Figure 1:**
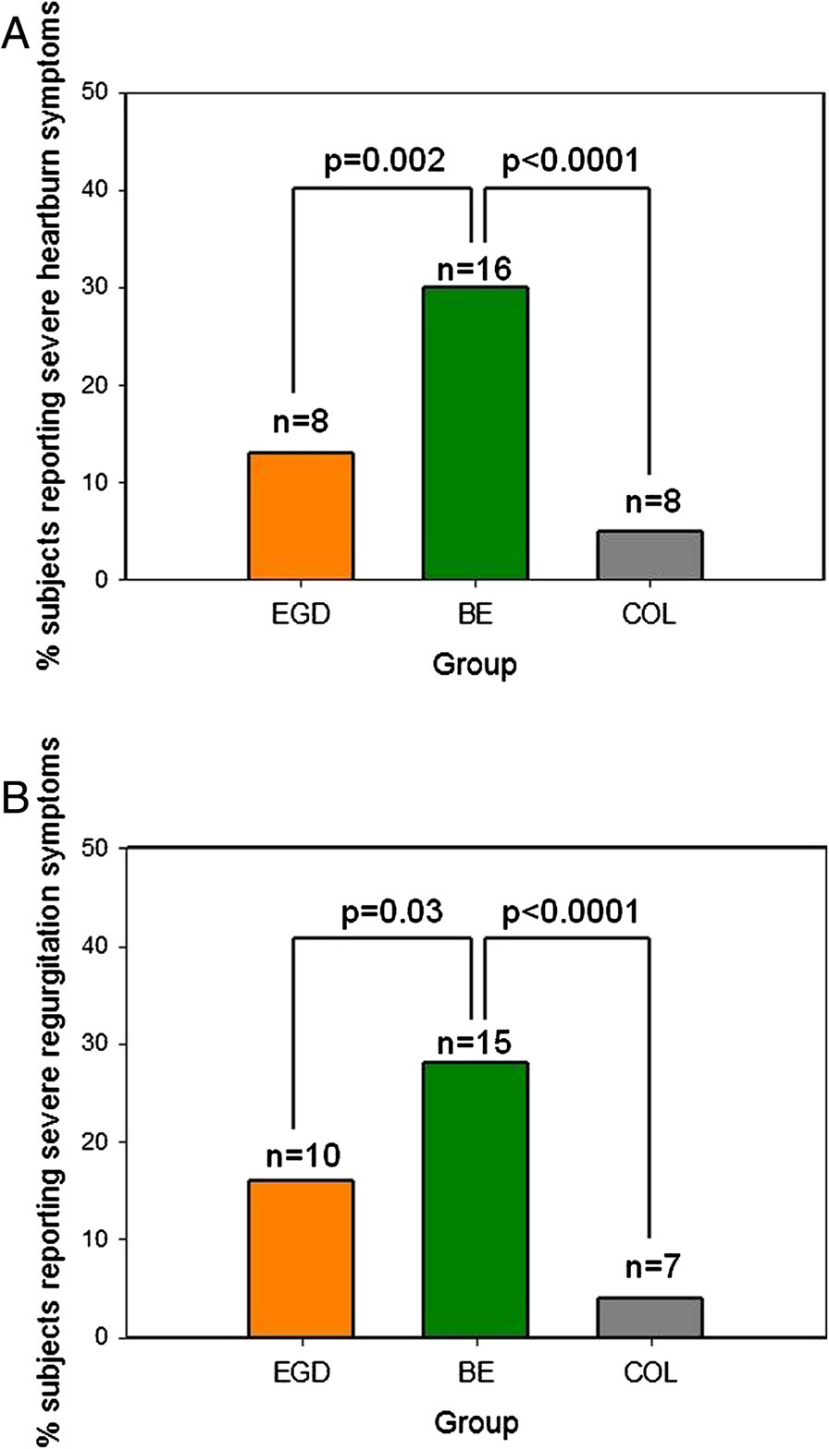
**Distribution of Severe Gastroesophageal Reflux Symptoms by Group.** Distribution of severe heartburn symptoms (**1A**) and severe regurgitation symptoms (**1B**) by group. Severe symptoms were defined as lifestyle-affecting. P values presented are results of Mantel-Haenszel chi-square tests.

Demographic characteristics, body mass indices, and GERD symptoms of the three comparison groups are presented in Table 1. Patients in the COL group were somewhat younger than the BE group (mean age, 57 years versus 63 years, p = 0.02). Although EGD patients had a significantly lower mean self-reported BMI than the BE group (27 kg/m^2^ versus 30 kg/m^2^), that in the COL group was not significantly different from the BE group. Not surprisingly, patients in the COL group were less likely to be male and white. BE patients were significantly more likely to report nocturnal regurgitation or nocturnal heartburn symptoms than either of the control groups (Table 1). In addition, BE patients reported more severe typical heartburn and regurgitation symptoms than either the EGD or COL groups (Figure 1A and 1B).

**Table 1:** Demographic characteristics, mean body mass index, and GERD symptoms among 3 comparison groups

	**BE (n = 54)**	**EGD (n = 62)**	**P value (BE vs. EGD)**	**COL (n = 171)**	**P value (BE vs. COL)**
Mean age, years (SD)	63 (11.1)	60 (11.7)	NS	57 (13.6)	0.001*
White (%)	53 (98.2)	59 (95.2)	NS	70 (41.9)†	<0.0001^¥^
Male (%)	38 (70.4)	36 (58.1)	NS	75 (43.9)	0.001°
Mean BMI, kg/m^2^ (SD)	30 (5.9)	27 (5.5)	0.03*	28 (5.6)	0.06*
Heartburn symptoms (%)	43 (79.6)	33 (53.2)	0.003°	53 (31.0)	< 0.0001°
Nocturnal heartburn symptoms (%)	27 (50.0)	16 (25.8)	0.01°	21 (12.3)	<0.0001°
Regurgitation symptoms (%)	42 (77.7)	38 (61.3)	0.06°	41 (24.0)	<0.0001°
Nocturnal regurgitation symptoms (%)	31 (57.4)	18 (29.0)	0.002°	18 (10.5)	<0.0001°

SD, Standard deviation

NS, Not significant

BMI, Body mass index

* Independent samples *t* test with equal variances

° Chi-square test

†Among respondents; 4 subjects did not respond to the question about race.

^¥^ Fisher’s exact test

Univariate logistic regression evaluating factors associated with BQ+ status revealed that BE was associated with an increased risk for OSA compared with COL controls but not EGD controls (Table 2). Within the BE group, 56% of patients scored at high risk for OSA on the Berlin Questionnaire (n = 30). In comparison, 42% of EGD patients (n = 26) were BQ+ (OR 1.73, 95% CI [0.83, 3.62]), and 37% of COL patients (n = 64) were BQ+ (OR 2.08, 95% CI [1.12, 3.88]). On univariate logistic regression among the BE and COL groups combined, age but not gender or race was also associated with an increased risk for OSA. Obese BMI based on self report, which does contribute to one symptom category of the Berlin Questionnaire, was also associated with markedly increased risk for OSA. A multivariate logistic regression model adjusting for age (Table 3) revealed that the odds ratio for the association between BE and BQ+ status was no longer significant at 1.84 (95% CI 0.98, 3.47). Upon comparing the multivariate logistic regression model containing age and BE status with the nested model containing age alone, the multivariate model did not provide significantly better fit by the likelihood ratio test, although the p value was minimally greater than 0.05 (chi-square test = 3.55, corresponding to a p value of 0.0595). Therefore, after adjusting for age, the association between BE and increased risk for OSA was no longer statistically significant. Likewise, a multivariate logistic regression model adjusting for age and BMI found no association between BE and BQ+ status (Table 3).

**Table 2:** Univariate analysis of factors associated with BQ+ status

	**Odds ratio**	**95% Confidence interval**
BE vs. EGD group	1.73	0.83, 3.62
BE vs. COL group	2.08	1.12, 3.88
Among BE and COL groups:		
Age	1.03	1.004, 1.047
Male gender	1.32	0.78, 2.25
White race (vs. non-white)	1.11	0.65, 1.90
BMI		
Normal	1.0	
Overweight	2.37	1.03, 5.46
Obese	16.8	7.30, 38.76

**Table 3:** Multivariate analysis of factors associated with BQ+ status among BE and COL groups

	**Odds ratio**	**95% Confidence interval**
Model containing age and BE status	1.73	0.83, 3.62
Age	1.02	1.00, 1.04
BE	1.84	0.98, 3.47
Model containing age, BE status, and BMI		
Age	1.03	1.00, 1.05
BE	1.51	0.72, 3.15
BMI		
Normal	1.0	
Overweight	2.32	0.99, 5.43
Obese	16.77	7.1, 39.42

**Figure 2:**
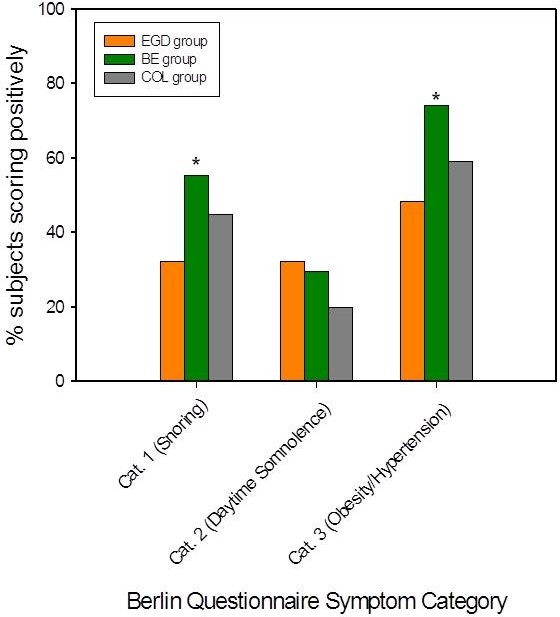
**Distribution of Positive Responses for each Berlin Questionnaire Symptom Category by Group.** Distribution of positive responses for each Berlin Questionnaire symptom category by group. Category 1 was positive if the subject had persistent snoring symptoms. Category 2 was positive if the subject had persistent daytime somnolence and/or drowsiness while driving. Category 3 was positive if the subject had a body mass index ≥ 30 kg/m^2^ or history of hypertension. BE, Barrett’s esophagus. EGD, esophagogastroduodenoscopy control group. COL, colonoscopy control group. Cat., category. * P ≤ 0.01 vs. EGD group.

Although the proportion of BQ+ patients was not significantly different between the BE and EGD groups, BE patients were more likely to score positively for 2 of the 3 symptom categories on the Berlin Questionnaire: snoring symptoms (56% for BE versus 32% for EGD, p = 0.01) and the presence of obesity/HTN (74% for BE versus 48% for EGD, p = 0.005). Figure 2 displays the proportion of subjects scoring positively for each symptom category of the Berlin Questionnaire among the 3 groups. However, within self-reported BMI strata (normal, overweight, or obese), there was no significant difference in snoring symptoms between the BE and EGD groups, suggesting that differences in BMI could explain the initial differences in snoring symptoms between the 2 groups. When BE patients were compared with the COL group, the proportion of subjects scoring positively for snoring symptoms or daytime somnolence did not differ significantly (56% for BE versus 45% for COL, p = 0.18 for snoring symptoms; 29% for BE versus 20% for COL, p = 0.13 for daytime somnolence/drowsiness while driving). BE patients were more likely to score positively for the 3^rd^ BQ symptom category, presence of obesity/HTN, although this did not reach statistical significance (74% for BE versus 59% for COL, p = 0.05).

Because COL patients who were not getting upper endoscopy served as controls and could potentially have been misclassified, we performed a sensitivity analysis to evaluate the extent to which misclassification could have affected our results. A previous study of 961 colonoscopy patients undergoing upper endoscopy revealed that 6.8% had Barrett’s esophagus; among colonoscopy patients with history of heartburn symptoms, 8.3% had Barrett’s esophagus, compared with 5.6% among those with no history of heartburn symptoms [6]. We therefore assumed that 7%, or 12, patients in the COL group in the current study could have been misclassified and actually had Barrett’s esophagus. Assuming that the BQ+ rate among these misclassified patients was 1) the same as that in the original COL group (a conservative estimate) versus 2) the BQ+ rate in the original BE group, then the odds ratio would be 1.75 (95% CI 0.98, 3.13) for the former scenario, versus 2.28 (95% CI 1.27, 4.10) for the latter scenario. In all likelihood, the true odds ratio probably lies somewhere in between.

We explored the relationship between nocturnal GERD and BQ+ status among all patients in the study, including cases and controls. Among all patients with nocturnal heartburn, the rate of BQ+ was 58% (n = 37), compared with 37% (n = 83) among patients without nocturnal heartburn (p = 0.003). Among all patients with nocturnal regurgitation, 63% were BQ+ (n = 42), compared with 35% (n = 78) among patients without nocturnal regurgitation (p < 0.0001). We also examined the association between nocturnal GERD symptoms (defined as either nocturnal heartburn or regurgitation, or both) and scoring at high risk for OSA within each study group (Table 4). Within the BE and COL groups, BQ+ subjects were more likely to report nocturnal GERD, although only 23% of BQ+ subjects reported nocturnal GERD in the COL group. There was no association between nocturnal GERD and BQ+ status within the EGD group.

**Table 4:** BQ+ status within each study group by nocturnal GERD symptoms

	**BE group (n = 54)**		**EGD group (n = 62)**		**COL group (n = 171)**	
	**nGER+ (n = 34)**	**nGER- (n = 20)**	**nGER+ (n = 21)**	**nGER-(n = 41)**	**nGER+ (n = 26)**	**nGER- (n = 145)**
**BQ+ (%)**	23* (67.6)	7 (35.0)	10 (47.6)	16 (39.0)	15* (57.7)	49 (33.8)

nGER, nocturnal gastroesophageal reflux symptoms (heartburn or regurgitation)

* p = 0.02 by chi-square test comparing nocturnal GERD symptoms within study group

## Discussion

The majority of BE patients in our pilot study scored at high risk for OSA on the Berlin Questionnaire. After adjusting for age and BMI, the association between Barrett’s esophagus and scoring at high risk for OSA compared with the COL group was no longer statistically significant. Because older age and higher BMI are risk factors for OSA [30], these factors likely had a large effect on the study results because the BE group was older than the COL group and had a higher BMI than the EGD group. Patients with known OSA were excluded from our study; however, almost all of these patients came from the BE group which could have affected our results. In our study, 37% of COL patients scored at high risk for OSA, which is slightly greater than the 32%-36% rate previously described in primary care settings and a cardiology referral clinic [26,29]. Patients presenting for outpatient endoscopy at our hospital may be a higher risk population than patients in primary care settings, who in turn likely have a greater prevalence of OSA than the general population [31]. Nonetheless, the high rate of BQ+ status among BE patients in our study is striking. Barrett’s esophagus patients may be more likely to have obstructive sleep apnea than GERD patients without BE due to insulin/insulin growth factor pathways which may have a role in the development of BE [32]; obstructive sleep apnea is associated with insulin resistance [20]. Since our study was conducted, two other recent studies presented in abstract form have suggested that BE is associated with obstructive sleep apnea [33] or obstructive sleep apnea characteristics [34]. Taken together, these findings hold particular relevance because BE patients who undergo endoscopic surveillance or lengthier endoscopic ablative procedures may thereby be at increased risk for sedation-related complications. Benzodiazepines and opioids can depress respiratory drive, decrease upper airway reflexes, and decrease pharyngeal muscle tone [35,36]. Propofol can also lead to upper airway collapse [35,37]. These effects are particularly important for patients with obstructive sleep apnea due to redundant pharyngeal tissue and generally narrower airways [38].

In the current study, 44% of EGD patients scored at high risk for OSA. This high rate is not surprising since many of these patients were likely undergoing EGD for GERD symptoms, and previous studies suggest that GERD is associated with sleep complaints [15,16]. In addition, Siupsinskiene and colleagues reported in abstract form results from a prospective Lithuanian study in which 42 OSA patients underwent upper endoscopy regardless of whether they had upper GI symptoms. This study revealed that 83.3% had pathologic GI findings: 64.3% had hiatal hernia, 45.2% had erosive esophagitis, and 21.4% had histological esophagitis [39]. There was no significant correlation between OSA severity and frequency of endoscopic findings. Although there was no mention of Barrett’s esophagus as an endoscopic finding, BE presumably could have been masked by erosive esophagitis.

In our study, BE patients were more likely than either of the control groups to report nocturnal heartburn or nocturnal regurgitation. BE patients also reported more severe typical GERD symptoms than patients in either control group. These findings are consistent with previous studies demonstrating higher esophageal acid exposure in BE patients [40,41]. Within the BE and COL groups, nocturnal GERD was associated with BQ+ status, although the rate of nocturnal GERD was low (23%) among BQ+ subjects in the COL group. These findings raise questions of whether BE could be associated with obstructive sleep apnea through a higher incidence of nocturnal GERD or whether nocturnal GERD itself increases the risk of obstructive sleep apnea. Previous studies have been inconsistent in demonstrating a link between nocturnal GERD and BE [21-24,42]. The relationship between GERD and OSA is incompletely understood. Nocturnal GERD was associated with sleep complaints [16] or OSA symptoms [43] in large cohort studies, and continuous positive airway pressure improved GERD symptoms in OSA patients [17,18]. However, a recent small study suggested no difference in the number of nocturnal gastroesophageal reflux events as measured by pH monitoring and impedance among OSA patients without GERD, OSA patients with GERD, GERD patients without OSA, and healthy controls [44]. In addition, high-resolution manometry measurements in that study suggested that during OSA events, gastroesophageal junction and upper esophageal sphincter pressures actually increased—changes which would be expected to prevent rather than induce reflux events.

Our pilot study had several limitations. Our study was limited by the use of open-access colonoscopy patients who were not getting EGD as controls, since we assumed that those patients did not have Barrett’s esophagus. The decision to include the COL control group in our study was partially due to slow accrual of EGD controls. Based on our sensitivity analysis, misclassification could have potentially strengthened or weakened an association between BE and BQ+ status. In addition, the study was limited due to exclusion of patients with known OSA; since most of these patients had Barrett’s esophagus, this could have affected the study results. Our study was also limited because results of the Berlin Questionnaire were not confirmed with polysomnography. Previous validation of the Berlin Questionnaire in a surgical patient population demonstrated a sensitivity of 68.9%-87.2% at various apnea-hypopnea index cutoffs [28]. Our assessment of nocturnal GERD was based on patient self-assessment of symptoms rather than an objective measure such as esophageal pH monitoring. The Berlin Questionnaire is partially based on self-reported BMI, which can vary significantly and unpredictably from the true BMI value and can potentially be misleading in very muscular patients. Finally, a type II error might explain why the association between BE and BQ+ status disappeared after adjusting for age.

## Conclusions

In summary, the findings from this pilot study demonstrate that a high proportion of Barrett’s esophagus patients score at high risk for obstructive sleep apnea. This finding is particularly important because many BE patients undergo endoscopic surveillance, and a minority undergo lengthier endoscopic procedures for ablation of dysplasia. BE patients may therefore be periodically exposed to sedation with its inherent risks. Given the high proportion of BE subjects at high risk for OSA in our study, screening for OSA should be considered in BE patients. At the very least, raising awareness that BE patients are likely to score at high risk for OSA may facilitate the judicious use of opioids and sedatives, consideration for use of capnography during sedation for lengthier endoscopic ablative procedures, and more vigilant post-procedure monitoring in BE patients undergoing surveillance endoscopy. Our results also demonstrated that patients scoring at high risk for obstructive sleep apnea were more likely to report nocturnal heartburn or acid regurgitation symptoms. Our findings support a growing body of literature linking nocturnal GERD to sleep disturbances. Future studies could explore mechanistic links among Barrett’s esophagus, nocturnal GERD, and obstructive sleep apnea.

## Abbreviations

EAC: Esophageal adenocarcinoma; BE: Barrett’s esophagus; GERD: Gastroesophageal reflux disease; OSA: Obstructive sleep apnea; EGD group: Esophagogastroduodenoscopy control group; COL group: Colonoscopy control group; RDI: Respiratory disturbance index; BQ: Berlin Questionnaire

## Competing interests

The authors declare that they have no competing interests.

## Authors' contributions

LCC participated in the study design, acquisition of data, data analysis, interpretation of results, and manuscript writing. NS, SM, WS, and VK participated in acquisition of data and editing the manuscript. AC conceived of the study and participated in the study design, acquisition of data, interpretation of results, and editing the manuscript. All authors read and approved the final manuscript.

## Pre-publication history

The pre-publication history for this paper can be accessed here:

http://www.biomedcentral.com/1471-230X/13/82/prepub
